# Impact of swimming school attendance in 3-year-old children with wheeze and rhinitis at age 5 years: A prospective birth cohort study in Tokyo

**DOI:** 10.1371/journal.pone.0234161

**Published:** 2020-06-09

**Authors:** Makoto Irahara, Kiwako Yamamoto-Hanada, Limin Yang, Mayako Saito-Abe, Miori Sato, Yusuke Inuzuka, Kenji Toyokuni, Koji Nishimura, Fumi Ishikawa, Yumiko Miyaji, Tatsuki Fukuie, Masami Narita, Yukihiro Ohya

**Affiliations:** Allergy Center, National Center for Child Health and Development, Tokyo, Japan; Telethon Institute for Child Health Research, AUSTRALIA

## Abstract

**Background:**

In Japan, swimming school attendance is promoted as a form of therapy or as a prophylactic measure against asthma in young children. However, the putative beneficial effects have not been sufficiently verified.

**Objective:**

The aim of the present study was to clarify whether or not swimming school attendance at age 3 years affects the onset and/or improvement of wheeze and rhinitis at age 5 years.

**Methods:**

This study was a single-center, prospective, general, longitudinal cohort study (T-CHILD Study). Between November 2003 and December 2005, 1776 pregnant women were enrolled, and their offspring were followed up until age 5 years. Swimming school attendance at age 3 years and the presence of wheeze and/or rhinitis in the previous one year were examined using the International Study of Asthma and Allergies in Childhood (ISAAC) questionnaire. The relationship between swimming school attendance and wheeze and/or rhinitis was analyzed using multivariable logistic regression analysis.

**Results:**

Data on the 1097 children were analyzed. At age 3 years, 126 (11.5%) children attended a swimming school, and at age 5 years, the prevalence of wheeze was 180 (16.4%) while that of rhinitis was 387 (35.3%). Swimming school attendance at age 3 showed no significant relationship with the development of either wheeze (aOR 0.83, 95% CI (0.43–1.60) or rhinitis (aOR 0.80, 95% CI (0.43–1.60) at age 5.

**Conclusions:**

Swimming school attendance at age 3 years showed neither a preventive nor therapeutic effect on wheeze or rhinitis at age 5 years. There is thus no scientific evidence yet that swimming school attendance has a positive impact on the development of childhood wheeze or rhinitis.

## Introduction

A 2015 study reported that the prevalence of wheeze in Japanese children at age 6–8 years was 10.2% [1]. Our study group previously identified five phenotypes in the trajectory of wheeze in Japanese children [2]. Asthma, a typical cause of wheeze, can lead to asthma-related death [3]. Furthermore, childhood asthma was associated with an increased psychological burden and decreased quality of life in caregivers [4,5].

The same study reported that the prevalence of rhinoconjunctivitis in children at age 6–8 years was 18.7% and that the prevalence of nasal conjunctivitis had been increasing for ten years [1]. Allergic rhinitis usually decreases QOL and is associated with sleep disturbance and reduced daily activity [6,7]. Thus, wheeze and rhinitis are common symptoms in the clinical setting and their prevalence is high among preschool-aged children. To prevent the development of wheeze and rhinitis from a younger age, the risk factors for their development need to be identified.

Swimming is one of the most popular physical activities among children worldwide. Swimming school attendance by children is also common. In 2013, 41.2% of children in Japan attended a swimming school [8], in part reflecting the common belief that swimming is effective in preventing or improving the symptoms of allergic diseases such as asthma [9,10].

A cohort study in the United Kingdom reported that preschool-aged children taking swimming lessons had a lower prevalence of wheeze at age 7 years than those who did not [11]. An age-matched controlled study reported that swimming was effective in alleviating wheeze and reducing school absenteeism in 6 to 12–year–old children with asthma [12]. On the other hand, some reports found that swimming pool attendance increased the prevalence of asthma [13,14]. Among children sensitized to inhaled allergens, those who received swimming lessons had significantly more asthma symptoms than those who did not [13]. Furthermore, swimmers who spent a longer time in the pool were found to be at greater risk of asthma development [14]. A recent meta-analysis reported that swimming pool attendance at a young age did not influence the prevalence or control of asthma [15]. Most of the studies included in another meta-analysis were cross-sectional and did not fully elucidate the effects of swimming school attendance on wheeze and asthma [16]. These previous reports were only observational, and no randomized controlled trials have yet been done to examine the preventive effect of swimming on asthma.

With regard to rhinitis, two studies found that children who swam regularly had a low QOL due to rhinitis, and that swimming pool exposure exacerbated their rhinitis symptoms [17,18]. The main causative factor behind these negative effects is thought to be exposure to reactants generated by contact with chlorine and organic substances, such as sweat [17,19]. Seys et al. reported that some individuals exposed to trichloramine, a by-product of chlorine, had cough, dyspnea, and blocked or runny nose [20]. Carbonnelle et al. reported that exposure to chlorinated water for one hour increased airway epithelium permeability [21]. These reports contributed to the current theory that repeated exposure to chlorine during swimming might lead to wheeze and rhinitis exacerbation. However, as in the case of asthma, the influence of swimming school attendance at a young age on subsequent rhinitis has not been sufficiently studied.

The above-mentioned reports on the effects of swimming school attendance on wheeze, asthma, and rhinitis were not from Japan, and there are unfortunately no prospective longitudinal studies in Japan on this topic despite the widespread belief that swimming lessons improve or prevent allergic diseases. The present, prospective, longitudinal birth cohort study of the general Japanese population aimed to clarify the relationship between swimming school attendance at age 3 years and the prevention or improvement of wheeze and rhinitis at age 5 years.

## Material and methods

### Research design, setting, and participants

The present study, named the “The Tokyo Children’s Health, Illness and Development Study (T-CHILD),” enrolled 1776 pregnant women between November 2003 and December 2005 as a general birth cohort [2,22,23] and recruited 1550 offspring born to these women. The lifestyle and health status of the families and children at pregnancy and at ages 3 and 5 years were evaluated by a questionnaire addressed to the parents. This study was conducted with the approval of the Ethics Committee of the National Center for Child Health and Development (Approval number: 52) and complies with the Japanese ethical guidelines (MHLW) for medical research on humans and the Helsinki Declaration. Informed consent was obtained from all participants at recruitment.

### Study outcomes and exposures

Wheeze and rhinitis at age 5 years were the primary outcomes. The prevalence of wheeze and rhinitis in the previous one year was evaluated using the International Studies of Asthma and Allergies in Childhood (ISAAC) questionnaire at ages 3 and 5 years [24]. The presence of wheeze (current or ever) was based on a positive response to the question, “Has your child ever exhibited wheeze or whistling in the past 12 months?” or “Has your child ever had wheeze or whistling at any time in the past?” The presence of current or ever rhinitis was based on a positive response from the caregiver to the question, "Has your child experienced sneezing or a runny or blocked nose in the past 12 months when he/she did not have a cold or the flu?” or "Has your child ever experienced sneezing or a runny or blocked nose when he/she did not have a cold or the flu?” Swimming school attendance at age 3 years was determined by a positive response to the question, “When your child was 3 years old, did he or she receive swimming lessons?”

### Confounding factors

The confounding factors were sex, sibling(s), maternal history of allergic diseases (asthma, atopic dermatitis, and rhinitis), maternal education level, household income, birthweight, maternal age at delivery, pet ownership at age 3 years, environmental tobacco smoke exposure at age 3 years, television (TV) viewing time on weekends at age 3 years, BMI Z-score at age 3 years, and breastfeeding before age 6 months. These confounding factors were evaluated using a questionnaire.

### Bias and sample size

The sample size was not calculated due to the exploratory nature of the study. The present study enrolled all children who were able to be followed up until age 5 years to minimize sampling bias. Further, the medical chart data of neither the patients nor high–risk children were analyzed. The study cohort was recruited from the general population to minimize selection bias.

### Statistical analyses

The nominal variables of maternal educational level, household income, and the children’s TV viewing time on weekends were defined, respectively, as junior high school vs. high school or older, less than 4 million yen vs over 4 million yen, and less than 3 hours per day vs over 3 hours per day. The nominal background variables were analyzed using the χ2 test to compare children with or without swimming school attendance at age 3 years. Wilcoxon’s rank sum test was used to analyze continuous variables excluding the BMI Z scores at age 3 years. Student’s T test was used to analyze the BMI Z scores at age 3 years. For wheeze and rhinitis outcomes, all confounding factors (sex, birthweight, siblings, pet ownership, environmental tobacco smoke exposure at age 3 years, TV viewing time, household income, maternal education level, maternal age at delivery, maternal history of asthma, atopic dermatitis and rhinitis, breastfeeding before age 6 months, and BMI Z-score at age 3 years) were added to and adjusted for in multivariable logistic regression analysis. The adjusted odds ratios (aOR) and 95% confidence intervals (CI) were analyzed by multivariable logistic regression. P < 0.050 indicated statistical significance.

In addition, children who attended swimming lessons to improve their symptoms were stratified based on the presence or absence of ever wheeze or rhinitis at age 3 years. The relationship of current wheeze and rhinitis at age 5 years with swimming school attendance was analyzed using multivariable logistic regression after adjusting for the above confounding factors. Multiple imputation (MI) was used to generate 50 imputed datasets, then logistic regression analysis of the multiple imputed data was performed. All statistical analyses were conducted using SPSS 19.0 for Windows (SPSS Inc., Chicago, IL, USA).

## Results

### Participants

Table 1 shows the background characteristics of the 1,550 children enrolled at birth. At age 5 years, 1097 children (70.7%) had current wheeze or rhinitis; 126 children (11.5%) attended swimming lessons at age 3 years, 265 children (24.2%) had ever wheeze, and 299 children (27.5%) had ever rhinitis at age 3 years. In addition, 180 children (16.4%) had current wheeze, and 387 (35.3%) had current rhinitis at age 5 years.

**Table 1:** Characteristics of the study population.

	N	Total	%
**Subject characteristics**			
Sex, male	553	1097	50.4
Sibling (s), >0	451	1096	41.2
Birthweight, g, median, percentiles 25, 75	2965	2715, 3235	
**Environmental exposure**			
Pet ownership	187	1095	17.1
Environmental tobacco smoke at 3y	341	1086	31.4
TV viewing at 3y >3h on weekends	95	1091	8.7
Income <4,000,000 yen/year	93	1025	9.1
**Maternal characteristics**			
Maternal age, y, media, percentiles 25, 75	34	31, 36	
Maternal education level, low^a^	262	1052	24.9
Maternal asthma	156	1097	14.2
Maternal atopic dermatitis	163	1097	14.9
Maternal rhinitis	554	1097	50.5
Maternal asthma, atopic dermatitis or rhinitis	639	1097	58.2
**Health assessment of children**			
Breastfeeding before age 6 months	164	1079	15.20
BMI Z-score at 3y, median, percentiles 25,75	0.14	-0.45, 0.65	
**Evaluation at 3y and 5y**			
Swimming lessons at 3y	126	1095	11.5
Ever wheeze at 3y	265	1095	24.2
Ever rhinitis at 3y	299	1088	27.5
Current wheeze at 5y	180	1096	16.4
Atopic dermatitis	231	1095	21.1
Current rhinitis at 5y	387	1096	35.3

TV: television, 3y: age 3 years, 5y: age 5 years, 6M: age 6 months

^a^maternal education level, low, middle school, high or vocational school

### Characteristics of swimming school attendees and non-participants at age 3 years

To examine the effect of swimming school attendance, the children were divided into two groups based on the presence or absence of swimming school attendance at age 3 years. Table 2 compares the background factors in the two groups. There was no significant difference between the two groups in ever wheeze or rhinitis at age 3 years or current wheeze or rhinitis at age 5 years. There was also no significant difference in the history of wheeze, sex, birthweight, pet ownership, environmental tobacco smoke exposure at age 3 years, maternal age at delivery, maternal education level, maternal asthma, atopic dermatitis and rhinitis or BMI z-score at age 3 years. The percentage of children with more than 3 hours of TV viewing on weekends was significantly higher in the swimming school attendance group (15.1% vs. 7.9%). The percentage of children with a sibling(s), family income < 4 million, and breastfeeding before age 6 months was significantly higher in the non-swimming school attendance group at 42.8% vs. 29.4%, 10.1% vs. 1.7%, and 16.3% vs. 7.3%, respectively.

**Table 2:** Distribution of confounding variables between swimming and non-swimming groups.

	Non-swimming at 3y		Swimming at 3y		P value
	Number	%	Number	%	
Sex, male	484	49.9	69	54.8	0.309
Birthweight <2500g	111	11.5	14	11.1	0.900
Sibling(s) >0	414	42.8	37	29.4	0.004^a^
Pet ownership	171	17.7	16	12.7	0.162
Environmental tobacco smoke exposure at 3y	305	31.8	35	28.2	0.423
TV viewing at 3y >3hr on weekends	76	7.9	19	15.1	0.007^a^
Income <4,000,000 yen/year	91	10.1	2	1.7	0.003^a^
Maternal age > = 35	406	41.9	49	38.9	0.513
Maternal education level, low	233	25.1	28	23.3	0.682
Maternal asthma	137	14.1	19	15.1	0.776
Maternal atopic dermatitis	138	14.2	25	19.8	0.097
Maternal rhinitis	491	50.7	62	49.2	0.757
Maternal asthma,	565	58.3	73	57.9	0.937
atopic dermatitis or rhinitis					
Breastfeeding before 6m	155	16.3	9	7.3	0.009^a^
BMI Z-score at 3y	0.15	0.89	0.10	0.85	0.555
Ever wheeze at 3y	235	24.3	30	23.8	0.903
Ever rhinitis at 3y	270	28.1	29	23.0	0.227
Current wheeze at 5y	163	16.8	16	12.7	0.237
Current rhinitis at 5y	346	35.7	40	31.7	0.377

TV: television, 3y: age 3 years, 5y: age 5 years, 6M: age 6 months

Sex, birthweight, sibling(s), pet ownership, environmental tobacco smoke exposure at 3y, TV viewing at 3y >3h on weekends, income <4,000,000 yen/year, maternal age > = 35, maternal education level, maternal asthma, atopic dermatitis or rhinitis, swimming lessons at 3y, ever wheeze at 3y, ever rhinitis at 3y, current wheeze at 5y, current rhinitis at 5y, and breastfeeding before 6m were analyzed using the ϰ2 test; BMI Z-score at 3y was analyzed using Student’s t test.

^a^P < 0.05

### Relationship between swimming school attendance at age 3 years and wheeze and rhinitis at age 5 years

Table 3 shows the relationship between swimming school attendance at age 3 years and current wheeze at age 5 years. The aOR for swimming school attendance at age 3 years with wheeze at age 5 years was 0.83 (95% CI, 0.40, 1.60], and P<0.05. Table 4 shows the relationship between swimming school attendance at age 3 years and current rhinitis at age 5 years. The aOR for swimming school attendance at age 3 years with current rhinitis at age 5 years was 0.80 (95% CI, 0.43, 1.60). P<0.05.

**Table 3:** Relationship between swimming school attendance at 3y and current wheeze at 5y.

	Unadjusted OR		Adjusted OR^a^		Multiple imputation	
	OR (95% CI)	P value	OR (95% CI)	P value	OR (95% CI)	P value
Swimming lessons at 3y (-)	1		1		1	
Swimming lessons at 3y (+)	0.72 (0.41–1.25)	0.239	0.83 (0.43–1.60)	0.575	0.74 (0.42–1.30)	0.296

CI: confidence interval, OR: odds ratio, 3y: age 3 years, 5y: age 5 years

Statistical analysis was done using multivariable logistic regression analysis.

^a^OR adjusted for sex, birth weight, sibling(s), pet ownership, environmental tobacco smoke exposure at 3y, television(TV) viewing at 3y, income, maternal education level, maternal age, maternal asthma, atopic dermatitis or rhinitis, breastfeeding before age 6 months, and BMI Z-score at 3y.

**Table 4:** Relationship between swimming lessons at 3y and current rhinitis at 5y.

	Unadjusted OR		Adjusted OR^a^		Multiple imputation	
	OR (95% CI)	P value	OR (95% CI)	P value	OR (95% CI)	P value
Swimming lessons at 3y (-)	1		1		1	
Swimming lessons at 3y (+)	0.84 (0.56–1.24)	0.378	0.80 (0.43–1.60)	0.374	0.83 (0.55–1.25)	0.373

CI: confidence interval, OR: odds ratio, 3y: age 3 years, 5y: age 5 years

Statistical analysis was conducted using multivariable logistic regression.

^a^OR adjusted for sex, birth weight, sibling(s), pet ownership, environmental tobacco smoke exposure at 3y, television(TV) viewing at 3y, income, maternal education level, maternal age, maternal asthma, atopic dermatitis or rhinitis, breastfeeding before age 6 months, and BMI Z-score at 3y.

### Relationship between swimming school attendance and current wheeze at age 5 years in children with or without ever wheeze at age 3 years

Table 5 shows that there was no significant relationship between swimming school attendance at age 3 years and current wheeze at age 5 years regardless of whether the subject had ever wheeze at age 3 years (aOR, 0.65 (95% CI, 0.22, 1.92), aOR, 0.93 (95% CI, 0.23, 1.50)). The MI process yielded similar results (aOR, 0.58 (95% CI, 0.23, 1.50), aOR 0.86 (95%CI, 0.37, 1.98)).

**Table 5:** Relationship between swimming lessons at 3y and current wheeze at 5y in children with or without wheeze at 3y.

		Unadjusted OR		Adjusted OR^a^		Multiple imputation	
		OR (95% CI)	P-value	OR (95% CI)	P-value	OR (95% CI)	P-value
Ever wheeze by 3y (-)	Swimming lessons at 3y (-)	1		1		1	
	Swimming lessons at 3y (+)	0.58 (0.23–1.49)	0.258	0.65 (0.22–1.92)	0.439	0.58 (0.23–1.50)	0.263
Ever wheeze by 3y (+)	Swimming lessons at 3y (-)	1		1		1	
	Swimming lessons at 3y (+)	0.80 (0.36–1.75)	0.568	0.93 (0.35–2.59)	0.931	0.86(0.37–1.98)	0.715

CI: confidence interval, OR: odds ratio, 3y: age 3 years, 5y: age 5 years

Statistical analysis was conducted using multivariable logistic regression.

^a^OR adjusted for gender, birth weight, sibling(s), pet ownership, environmental tobacco smoke exposure at 3y, television(TV) viewing at 3y, income, maternal education level, maternal age, maternal asthma, atopic dermatitis or rhinitis, breastfeeding before age 6 months, and BMI Z-score at 3y.

### Relationship between swimming school attendance and current rhinitis at age 5 years in children with or without ever rhinitis at age 3 years

Table 6 shows that there was no significant relationship between swimming school attendance at age 3 years and current rhinitis at age 5 years regardless of whether the subject had ever rhinitis at age 3 years (aOR, 1.12(95%CI, 0.64, 1.98), aOR, 0.39 (95% CI, 0.14, 1.14)). The MI process yielded similar results (aOR, 1.03 (95%CI, 0.62, 1.70), aOR, 0.60 (95% CI, 0.26, 1.35)).

**Table 6:** Relationship between swimming lessons at 3y and current rhinitis at 5y in children with or without rhinitis at 3y.

		Unadjusted OR		Adjusted OR^a^		Multiple imputation	
		OR (95% CI)	P value	OR (95% CI)	P value	OR (95% CI)	P value
Ever rhinitis by 3y (-)	Swimming lessons at 3y (-)	1		1		1	
	Swimming lessons at 3y (+)	1.06 (0.66–1.72)	0.808	1.12 (0.64–1.98)	0.689	1.03 (0.62–1.70)	0.921
Ever rhinitis by 3y (+)	Swimming lessons at 3y (-)	1		1		1	
	Swimming lessons at 3y (+)	0.56 (0.26–1.21)	0.141	0.39 (0.14–1.14)	0.084	0.60 (0.26–1.35)	0.213

CI: confidence intervals, OR: odds ratio, 3y: age 3 years, 5y: age 5 years

Statistical analysis was conducted using multivariable logistic regression.

^a^OR adjusted for gender, birth weight, sibling(s), pet ownership, environmental tobacco smoke exposure at 3y, television(TV) viewing at 3y, income, maternal education level, maternal age, maternal asthma, atopic dermatitis or rhinitis, breastfeeding before age 6 months, and BMI Z-score at 3y.

## Discussion

This study found no relationship between swimming school attendance at age 3 years and current wheeze and rhinitis at age 5 years. Moreover, swimming school attendance at age 3 years did not improve wheeze or rhinitis symptoms at age 5 years. The strength of this study was its use of a prospective longitudinal birth cohort recruited from the general population in Japan, the first such study to be undertaken in the nation. Only a few, similar studies have been carried out to date. Thus, this report makes an important contribution to clarifying whether or not swimming, a popular sport among young children, has any effect on the prevention or improvement of childhood asthma and rhinitis.

Previous reports indicated that attendance at swimming school affected the incidence of asthma in children with atopy. Bernard et al. examined the prevalence of wheeze in adolescents (13–18 years of age) in terms of the total IgE >30 IU/ml and the cumulative time spent in a pool [25] and reported that subjects with more than 1000 hours in a pool had a significantly higher prevalence of wheeze than those with less than 100 hours. Andersson et al. reported an association between wheeze in children aged 11–12 years with once weekly swimming lessons and also showed that among children who were sensitized to inhaled allergens, those who received swimming lessons showed greater asthma exacerbation than those without swimming lessons [13]. Further, a cross-sectional study by Voisin et al. showed that among children with a history of bronchitis, those who had received swimming lessons before age 2 years (mean age: 5.7 years) had a higher prevalence of asthma and allergic rhinitis [26]. However, they found no significant difference between subjects with low total IgE (non-sensitized group) and those without bronchitis. These findings differed from our own, suggesting that children with atopy may be adversely affected by swimming pool exposure.

It has been suggested that chlorine, which is used to sterilize pool water, may affect the lung epithelium [27]. In addition, it has been shown that elite swimmers have different outcomes for wheeze and rhinitis due to differences in the duration of chlorine exposure [16,25]. As noted above, Bernard et al. demonstrated a significant difference in the prevalence of wheeze in children with a cumulative swimming time exceeding 1000 hours than in those with less than 100 hours [25]. Surda et al. examined the effect of rhinitis on the QOL of swimmers aged 10 years or older and found that the QOL was lower in an elite swimmer group than in a non-elite swimmer group [17]. Our study included children with swimming school attendance at age 3 years, who may not have had sufficient exposure to chlorinated pool water to manifest any significant relationship with asthma or rhinitis. This difference in exposure levels may explain the discrepancies in the findings.

Other studies have indicated that swimming had the positive effect of decreasing wheeze due to improved lung function [11,28]. Font-Ribera et al. studied children with a history of wheeze at age 7 years and reported that the forced vital capacity (FVC) and the forced expiratory volume% in one second (FEV1) were higher in children who swam than in those who did not [11]. Yilamz et al. demonstrated that swimming for eight weeks had a positive effect on respiratory muscle strength and lung function in adults [28]. In the present study, pool attendance had no effect on wheeze or rhinitis at age 5 years in children with a past history of wheeze or rhinitis. The previous studies enrolled adults; thus, the effects of swimming may differ depending on the age of the swimmer.

In fact, some previous studies have also indicated the absence of a relationship between swimming school attendance at a young age and wheeze and rhinitis at around age 5 years. A study by Font-Ribera et al. comparing the development of wheeze at age 6–12 years in children who attended swimming school up to age 2 years and those who did not found no significant difference between the groups [29]. In another study, the same authors also reported the absence of any relationship between swimming attendance up to age 4 years and the development of hay fever symptoms at ages 7 and 10 years [11]. Andersson et al. reported the absence of any relationship between a history of swimming before age 1 or 2 years and the development of wheeze by school-age [30]. The findings of these studies were thus in line with our own. As noted above, swimming pool exposure may differ by age; it is possible that in the general population, swimming school attendance up to age 3 years may play a limited role in wheeze and rhinitis development in preschool children.

The present study has several limitations. First, the cumulative time spent in a pool, frequency of swimming school attendance, and chlorine concentration in the pool water were not taken into account. However, as mentioned above, the majority of children attending a swimming school at age 3 years were not likely to have spent large amounts of time in a pool. Moreover, it is unlikely that the chlorine concentrations varied widely given the Ministry of Health, Labour and Welfare’s specifications (issued in May 2007) as to the amount of chlorine to be added to pool water as a disinfectant [31]. Thus, there is unlikely to be a large bias in chlorine exposure in preschool children compared to school-aged children. Second, information on treatments for wheeze and rhinitis were not considered. Finally, sub-group analysis of children with IgE sensitization was not performed. However, the cohort enrolled in the present study was drawn from the general population, and the results are therefore likely to be representative of the general population in Japan.

The present study longitudinally assessed the causal relationship between swimming pool exposure at a young age and wheeze and rhinitis development in preschool children recruited from the general population. Swimming school attendance by age 3 years did not affect the onset or improvement of current wheeze or rhinitis symptoms at age 5 years. Although previous studies indicated that swimming school attendance had either deleterious or beneficial effects on wheeze and rhinitis, the present study failed to find any such effects in the general Japanese population. Future studies investigating the effects of swimming on children of different ages and levels of sensitization in terms of duration and frequency of exposure to chlorinated water, etc., are desirable.

The American Academy of Pediatrics stated that the scientific basis for the effectiveness and safety of swimming school participation up to age 4 years is unclear, and concerns have been raised about the risk of drowning. Cases of convulsion and hypoxemia caused by water intoxication during swimming lessons have been reported [32,33]. In the United States, drowning was reportedly the most frequent cause of accidental death in children between the ages 1 year and 4 years [34]. Allergies are thus not the only risk inherent in swimming school attendance in young children.

## Conclusions

Exercise can clearly benefit children's growth and development and should be encouraged; however, swimming school attendance at age 3 years showed neither a preventive nor therapeutic effect on wheeze or rhinitis at age 5 years. The current study found that swimming school attendance for prevention and therapy of wheeze and rhinitis at a young age should be de-emphasized due to the lack of any consistent scientific evidence indicating that it has a preventive or curative effect on asthma or rhinitis.
